# Structural factors shaping health-seeking behaviour among breast cancer survivors in the Gaza Strip: A qualitative study utilising the right to health framework

**DOI:** 10.1371/journal.pgph.0003718

**Published:** 2025-04-21

**Authors:** Walaa Ammar-Shehada, Benjamin Bouquet, Piet Bracke, Melissa Ceuterick

**Affiliations:** 1 Health and Demographic Research, Ghent University, Ghent, Belgium; 2 Population Health Sciences Institute and School of Law, Newcastle University, Newcastle, United Kingdom; Rwanda Military Hospital, RWANDA

## Abstract

This paper aims to analyse the enabling and disabling factors for health-seeking behaviour among Palestinian breast cancer survivors in the Gaza Strip. Specifically, it investigates how the availability of and accessibility to healthcare services influence health-seeking behaviour during the detection and treatment stages. The core components of the Right to Health—accessibility, availability, acceptability, and quality serve as the theoretical framework for this study. Data were gathered through forty semi-structured interviews, predominantly with breast cancer survivors (38 participants). The collection of this data occurred between September 2020 and April 2021. These interviews were analysed using a framework analysis approach. The study’s findings reveal that a portion of the participants were initially reluctant to seek healthcare before their diagnosis as they perceived more barriers than threats, combined with other priorities of family and work stepping in and due to lack of clear guidance and information on the starting point and overall procedures. However, following the confirmed diagnosis, there was a solid sense of determination among the participants to obtain necessary care, whether within Gaza or elsewhere, due to the perceived threat to life. The engagement in post-treatment follow-up was strongly affected by existing barriers again, such as the economic status of each individual. It was frequently driven by peer and family support, mainly through guidance and financial assistance, which played a crucial role in bridging affordability gaps and addressing limited access to information on specific services. The results indicate that participants actively sought healthcare once a life-threatening diagnosis was confirmed. However, barriers to the Right to Health components significantly influenced their health-seeking behaviour both before diagnosis and during post-treatment periods. These findings highlight the crucial impact of socioeconomic and health system-related factors such as accessibility and availability of health services on the health-seeking behaviour of patients in Gaza.

## 1. Introduction

Cancer ranks as the second leading cause of mortality in the occupied Palestinian territory (oPt) [1]. Breast cancer (BC), constituting 18.6% of all cancer cases in Palestine, is the predominant cancer among women and girls [2]. The oPt consists of two geographically disconnected parts: the Gaza Strip (GS) and the West Bank (WB). The whole territory has been under Israeli occupation for more than 57 years and the GS has endured over 18 years of land, sea, and aerial blockade since 2007. Consequently, public healthcare across the oPt, particularly in Gaza, faced critical shortages in the availability of essential medical equipment, supplies and human resources. Before the ongoing escalation of violence since 7th of October 2023, on average, 40% of essential medicines and 18% of essential medical supplies at Gaza’s central drug store were below a month’s stock in 2022 [3]. These shortages have led to a high reliance on medical referrals outside the Ministry of Health (MoH) facilities, accounting for nearly 37.5% of the total Palestinian MoH expenditure in 2021 [3]. In July 2023, approximately 26% of all MoH referrals were for oncology [4]. However, the systematic delay and denial of patient travel permits severely impact patients’ health [5]. In 2022, oncology patients represented 35% of total patient permit applications, of which only 67% obtained approval to exit Gaza for treatment [6].

The low survival rate of BC in the GS can be attributed to advanced-stage diagnoses and a lack of timely access to treatment services [1]. Structural barriers significantly hinder patients’ access to essential services, including the unavailability of specific treatment protocols locally, and unreliable treatment pathways outside Gaza [7].

Since October 7, 2023, all previous arrangements for referrals have ceased, leaving cancer patients without access to specialised treatment outside Gaza. Moreover, access to healthcare within Gaza has become severely restricted. In most governorates, access to services is now non-existent, primarily due to life-threatening Israeli evacuation orders from many areas and attacks on healthcare. These attacks have resulted in more than half of the hospitals shutting down, and the remaining ones are only partially functioning, primarily to provide trauma care [8].

This study explores women’s behaviour in seeking health care in uncertain circumstances, both before and after diagnosis. Advanced-stage diagnosis in Gaza is partly attributable to gaps in organised screening programs [9]; to low knowledge about self-examination practice, symptoms, and risk factors of BC [10]; and to embarrassment and fear of diagnosis [9]. Additionally, different socio-demographic groups of women in Gaza exhibit varying health-seeking behaviors, influenced by individual and household decisions, community norms, expectations, and provider characteristics [11]. Ammar-Shehada and Bracke (2023) highlighted inequalities in the stage of BC diagnosis in Gaza based on factors such as age, marital status, education, employment, and refugee status [12]. Health-seeking is not straightforward behaviour and was approached by different theories. One of them is the Health Belief Model [13] that focuses on the notion of ‘perceived susceptibility,’ which relates to the perceived likelihood of developing a particular condition. This, along with ‘perceived severity,’ contributes to forming a ‘perceived threat’ regarding the said condition. The findings of reviewing 29 health behavioural model-related investigations found that perceived susceptibility is more associated with preventive health conditions, and perceived threat, and accordingly, perceived benefits were more associated with the sick-role behaviour. ‘Perceived barriers’ were found to be the most powerful dimension across behaviour in both cases [13]. However, the model largely ignores the contextual features that enable or limit health behaviour. Its main focus is rational intentional behaviour over routinized behaviour. This paper examines the structural factors that affect the *health-seeking behaviour* of BC survivors, utilising the *Right to Health (RTH)* core components as an analytical lens, to see how the women-seeking behaviour is shaped by the wider institutional factors. The *RTH* core components (also referred to as “essential elements”), outlined by the Committee on Economic Social and Cultural Rights in general comment 14 [14], are fundamental to the realisation of the highest attainable standard of health and well-being. Those core components comprise availability, accessibility, acceptability, and healthcare quality. Availability refers to sufficient health facilities, goods, and services for all. Accessibility involves ensuring these facilities and services are universally reachable, considering non-discrimination, physical, economic, and informational aspects. Acceptability requires health services to respect medical ethics and cultural sensitivities, focusing on people-centred care and adhering to international ethics standards. Quality extends to ensuring clean water and sanitation in health facilities, as well as ensuring facilities and services are scientifically and medically sound [14].

The Committee emphasises that the RTH extends to safeguarding social conditions of life that influence health, encompassing political, economic, and legal dimensions [14]. Furthermore, a human rights-based approach to health seeks to uphold fundamental human rights principles of participation, empowerment equity, non-discrimination, accountability and the rule of law in healthcare delivery [14–16].

Given the pressing challenges and structural barriers in the oPt, alongside the varied behavioral patterns depending on the perceived threat to life, this study underscores the importance of analyzing these variations from the perspective of duty holders. Thus, this study aims to investigate factors, both facilitating and hindering, that impact the health-seeking behaviour of BC survivors in the GS as presented in their personal accounts. Specifically, it aims to address the question: How do institutional factors influence health-seeking behaviour during both the detection stage and the treatment journey among Palestinian BC survivors?

## 2 Data and methods

### 2.1 Ethics statement

The Helsinki Committee for Ethical Approval of the Palestinian Health Research Council provided written approval for this research (PHRC/HC/730/20). The MoH granted a facilitation letter (number 527744) to collect data related to the research from health facilities. Approval was obtained from NGOs who supported the recruitment of participants. Informed verbal consent was obtained from the interviewees before starting the audio recording.

### 2.2 Study design and settings

This study used a qualitative design due to the understudied nature of the topic and the need to deeply explore the personal narratives of BC survivors in Gaza, capturing the complexities of their experiences before and after illness. Individual semi-structured interviews [17] were used to capture the participants’ narratives about their diagnosis and treatment journey and enabled us to gather rich, detailed data, essential for understanding the intricate process of health-seeking behavior in a conflict-affected area. The first author (WA-S) collected the data through interviews between September 2020 and April 2021. The first author carried out visits to the oncology department of Al-Rantisi Governmental Hospital, which was the only hospital for treating cancer patients during COVID-19 at the time of data collection. Thirty-eight survivors were recruited using a convenient sampling strategy by employing several recruitment techniques, i.e., (i) contacting women who had agreed to share their contact information during visits to the oncology departments, (ii) through NGOs working with these patients, (iii) this paper was part of mixed-method research that incorporated a survey [12] as one of the data collection tools. Participants who had willingly shared their information during the completing the survey to be approached for the qualitative segment were contacted. The selection criteria were diagnosis of BC and living in Gaza during diagnosis and treatment. As more women expressed interest in participating in the qualitative part, a purposive sampling technique was followed to ensure diversity among participants, reaching out to individuals from various socio-demographic categories and at different stages of their treatment/recovery. Interviews were conducted with women of varying ages and illness stages, ensuring diversity in geographical locations and marital statuses. Efforts were made to ensure adequate representation of refugees, given that approximately 74% of the general population in Gaza at the time of the study was registered as refugees [18]. This process continued until data saturation was achieved, meaning that patterns observed in the narratives of experiences could lead to conclusions among the overall group of patients or subgroups within them. In addition, two semi-structured interviews were conducted with a patient’s mother and a BC activist/ manager of one of the NGOs serving cancer patients. All interviews were conducted in Arabic. Quotes that were relevant to the emerging themes were highlighted, and the final ones that were to be used in the paper were translated into English by the first author (WA-S). Interviews were conducted face-to-face, at home or at NGOs, through a video/telephone call based on COVID-19 physical distancing measures enforced at the time of the interview and based on the interviewee’s preference. All interviews were audio recorded after obtaining informed oral consent from interviewees; and then, fully transcribed in Arabic with the help of a research assistant. The first three interviews were fully translated into English by (WA-S) and shared with the co-authors (PB) and (MC) to validate the interviewing topic guide and for general feedback.

The topic guide included personal information, health conditions and history of illness and treatment, social and economic context, family structure, place of residence, support received, challenges and stressful experiences, and changes and coping after illness at different levels.

### 2.3 Analytical framework and coding

Framework analysis is fundamentally a comparative method of thematic analysis that utilises a structured framework derived from both inductive and deductive approaches [19]. This framework serves as a systematic tool for conducting cross-sectional analysis through a blend of data description and abstraction. In this paper, the analysis was guided by the RTH framework.

The RTH framework comprises four interconnected core components: availability, accessibility, acceptability, and quality (AAAQ) [14]. The impact of availability on women’s seeking behaviour was assessed by examining whether sufficient health facilities and services were accessible to all, and how the presence or absence of these services influenced women’s motivation to seek healthcare. Accessibility was evaluated to understand how easily women could access and utilize services, considering factors such as discrimination, physical barriers, economic constraints, and information availability in their decision-making process. Acceptability was analysed by assessing adherence to medical ethics and cultural sensitivities, with a focus on patient-centred care and compliance with international ethical standards. Quality was examined by assessing how facilities and services adhered to scientific and medical standards. This guided the analysis to investigate treatment adherence following diagnosis and its effect on healthcare-seeking behaviour both before and after confirmation of diagnosis.

Data were manually coded on paper by the first author using the AAAQ framework to ensure a detailed and nuanced understanding of the participants’ experiences. This manual coding process involved an iterative approach, where data were carefully read and assigned to thematic categories based on emerging themes and patterns. Special attention was given to subcategories. For example, under accessibility- non-discrimination, physical accessibility, economic accessibility (affordability), and information accessibility, were tracked systematically throughout the analysis. This was followed by live meetings with the other authors to go through the preliminary. This stage involved collaborative discussions aimed at validating and refining the codes and their interpretations. This validation process significantly enhanced the reliability and validity of our findings by incorporating diverse perspectives and expertise.

## 3. Results

**Fig 1:**
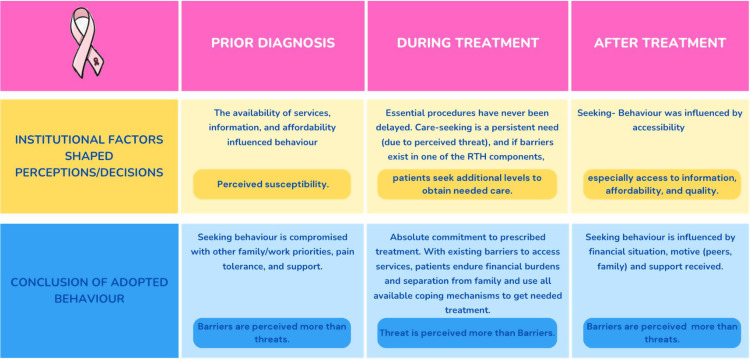
Summary of barriers and facilitating factors of health-seeking behaviour among breast cancer patients in Gaza.

The study investigates how structural arrangements of health systems influence health-seeking behaviour, examining differences before diagnosis, during treatment, and post-treatment (summarised in Fig 1). Participants displayed mixed responses in seeking care post-symptom onset, with delays often due to work, study, family commitments, lack of information or financial resources. Post-diagnosis, all participants proactively sought necessary treatment and attempted to utilise available coping mechanisms to overcome barriers to accessing treatment. Among those who recovered, adherence to follow-up varied, with women participating in peer support groups showing full compliance, while others were influenced by social factors like poverty. The study’s results will be presented through the lens of the four core components of RTH: AAAQ, and their impact on health-seeking behaviour at various stages: pre-diagnosis, during treatment, and post-treatment follow-up.

### 3.1 Study participants

Table 1 shows a summary of participant survivors’ characteristics. Women were between 29 and 72 years old with an average of 47.6 years old. 27 were married (including two abandoned and two separated); five were divorced; three were widowed, and three were single. 25 (out of 38) were refugees. Nine attained primary/preparatory education, 20 attained secondary education and nine post(graduate) education. Five had income through employment, retirement, or volunteering, and 33 were unemployed (including three who lost their job due to BC).

**Table 1:** Characteristics of participants (N = 38).

Marital status	
Married	27
Divorced	5
Widowed	3
Single	3
Age	
26–40 years	12
41–50 years	7
51–65 years	17
65+ years	2
Refugeehood	
Refugee	25
Non-refugee	13
Education	
Primary/Preparatory (1–9 years of education)	9
Secondary (10–12 Years of education)	20
post(graduate) (>12 years of education)	9
Employment	
Employed, volunteer, retired	5
Unemployed	33

### 3.2 Availability

Participants emphasised that the lack of medical equipment, pharmaceuticals, and specialists in the healthcare system led to increased waiting times and out-of-pocket expenses as reflected in quotations 1 and 2. For essential treatment protocols, women often opt to pay for quicker service in the private sector when faced with long waits in the public sector. Those who could not afford this turned to coping mechanisms like borrowing money from relatives or selling personal items like gold to cover necessary costs. Patients constantly had to weigh the options of undergoing immediate but radical procedures in Gaza against the uncertain wait for a referral and exit permit for treatment outside Gaza. This often led to less favourable but more expedient decisions to increase their chances of survival. Nevertheless, for cases who needed radiotherapy, waiting to get referred and obtain a travel permit – if successful- was the only way as radiotherapy is not available in Gaza, neither in the public, private or NGO sectors.

In the post-treatment phase, with the immediate threat to life reduced, there was a tendency to delay purchasing supplements and scheduling periodic imaging, reflecting a shift in urgency and financial prioritisation once out of the critical treatment phase.

*Quotation 1: “It has been four months since I took my prescribed monthly [Zoladex] injection. It is important to make the medication available. It must be taken regularly.” (interviewee #17)*

*Quotation 2: “We keep moving between hospitals. Lab tests here and there. It should be one place for cancer service… lab tests are often unavailable, and we are told to take them outside the hospital. The gland test is never found in the hospital, always in a private lab, and I pay about 100 ILS [27 USD] … We need one place to make all our tests and to pay a reasonable share of cost” (interviewee #23)*

### 3.3 Accessibility

The data reveals that after experiencing symptoms like pain or noticing signs of disease such as a lump, many women delay seeking medical attention due to other pressing events, including exams and family occasions. This behaviour was observed regardless of the women’s educational backgrounds. Even several university-educated women reported tolerating pain and postponing visits to health facilities.

However, upon receiving a confirmed diagnosis post-biopsy, these women promptly started to take serious action. They actively pursue the necessary treatments, often at their own expense, recognising that delays, especially in the public healthcare sector, could exacerbate their condition. This shift in urgency highlights the impact of a confirmed diagnosis on health-seeking behaviours.

*Quotation 3* shows an example related to two key aspects of accessibility in healthcare: physical accessibility and non-discrimination. It illustrates a scenario where a patient benefiting from personal connections (such as a relative in the health sector) experiences expedited referral processes, contrasting with others who lack such advantages. This disparity underscores issues of inequity in access to healthcare, mainly due to high demands on services that are unavailable locally. Other participants reported a willingness to endure hardships, like prolonged stays away from home and separation from family, for necessary treatment outside Gaza, even if no companion was granted a permit to travel with them. These challenges are compounded by the uncertainties and delays brought about by the COVID-19 pandemic, adding further stress to their health-seeking journey. This situation reflects the complex interplay of physical accessibility and the principle of non-discrimination in healthcare access, particularly during crisis periods.

*Quotation 4 and 5* exemplify the issue of affordability in healthcare. Throughout conversations with participants, financial hardship emerged as a significant concern for cancer patients in Gaza. Despite being exempted from major treatment costs at government hospitals or referred institutions, the associated expenses for medications and transportation pose a substantial burden, particularly for impoverished women. A common coping mechanism involves obtaining essential medications on credit from local pharmacies, a widespread practice in Gaza. Another strategy is borrowing money from relatives to cover the expenses associated with travel for treatment.

*Quotation 6* highlights issues with accessibility to information in healthcare. Participants’ narratives revealed a significant lack of coordination among health providers in government sectors, private entities, or NGOs. This disjointed system leaves patients without a reliable source for addressing side effects and follow-up care concerns. The absence of coordinated care and internal referrals exacerbates the challenge of timely access to crucial health information. However, participants noted better information access through peer networks, where they could learn from the experiences of previous survivors or through family histories of cancer. Additionally, cancer awareness activities served as valuable platforms for gathering information.

*Quotation 3: “Doctors informed me that I must be referred outside Gaza because having surgery in Gaza will not be up to the needed level. They will remove more things than needed because there is no possibility of selecting the cells to be removed preciously, so I have waited 40 days to get my referral. I did not wait like any patient, as we know people who pushed to get my referral fast; otherwise, the cancer would have spread more. I got it after 40 days, which is not too long a waiting time [compared to other patients].” (Interviewee #4)*

*Quotation 4: “Medications are overwhelming expenses. I swear to God, when I was going to chemotherapy sessions, I had no money to pay for transportation. We had debts from everybody to do my surgery… I was surviving on a daily wage, and then I accumulated unexpected debts from treatment-related expenses.” (interviewee #19)*

*Quotation 5: “I worry about how I can travel,” said a 43-year-old refugee survivor, tears streaming down her face, “I will stay indebted to people.” (interviewee #7)*

*Quotation 6: “The problem is that doctors do not tell me I need to do physiotherapy after surgery. We got to know this from NGOs, and we got to know other information about nutrition and physical activities as well. I knew these organisations one year after I finished my treatment protocol.” (interviewee #11)*

### 3.4 Acceptability

Most participants expressed less trust in the local health system than in the WB or Jordan oncology care. Consequently, they readily accepted referrals outside the local governmental facilities when available. However, when referral was not an option, they utilised local services, often preferring the private sector for its privacy and confidentiality, though affordability was a significant issue for many.

In terms of acceptability, patients faced challenging situations, especially when travelling to hospitals outside Gaza. At checkpoints like Beit Hanoun in the north of the Gaza Strip, invasive procedures such as strip searches were sometimes required, with refusal potentially leading to the denial of travel. This situation placed patients in a difficult position, having to choose between their dignity and their health needs, as reflected in *Quotation 7.*

In summary, life-saving procedures were prioritised, often overriding personal preferences. For many Gaza patients, acceptability was seen as a luxury, secondary to the urgent need for treatment and survival.

*Quotation 7: “They told us about the Basmet Amal Association that helps pay for transportation. I went to the checkpoint [to exit Gaza for the destination hospital] but was delayed about 3 hours in the security search, so the bus [of the association] left. I did not plan for this, and I had to pay 600 ILS [166 USD] for transportation for me and my father [as a companion]. We left our home early in the morning, but that day, we did not eat or drink later during the day because we only had 50 ILS [13 USD] left for the trip [which could last from days to weeks].” (Interviewee #19)*

### 3.5 Quality

The study identified several key subthemes that participants consistently mentioned. One of the most prevalent was the short time of patient-doctor contact in hospitals, which many felt was insufficient. Additionally, there were significant concerns about inadequate pain management. Another central theme was the experience of uncertainty and a lack of accountability within the healthcare system. A notable number of cases (11 out of 38 patients) reported delays in receiving a diagnosis after initially presenting symptoms. For some, the confirmation of their diagnosis took months or even up to a year. This delay in diagnosis was a source of frustration and overlapped with trust issues. Many women felt compelled to consult multiple health providers to ensure an accurate diagnosis, reflecting a lack of confidence in the initial healthcare responses they received. This practice results in additional time and financial consumption.

A significant theme that emerged from the study was the challenge of pain management as well as the absence of psychological support during treatment. Women reported a solid aversion to attending their subsequent chemotherapy sessions due to the intense pain experienced during and after the treatment. This dread of the anticipated pain was a common sentiment. However, despite this discomfort and fear, it was noteworthy that these women consistently adhered to their treatment schedules. They neither skipped nor delayed their chemotherapy sessions, underlining their commitment to the treatment process.

## 4 Discussion

### 4.1 Accessing healthcare in protracted conflict-affected areas

Local availability of oncology services is already challenging, which is why accessibility is examined within Gaza and outside Gaza in the paper. Access to health care is a multidimensional concept that has been defined as the “degree of fit” between a patient’s socioeconomic characteristics, the health system, and the health services organization [20]. Existing literature emphasises the significant role of financial difficulties as a substantial impediment to seeking medical attention for BC [21,22]. Previous studies have identified economic hardships, fear, and a scarcity of cancer treatments/equipment as crucial factors limiting access to BC care [23]. This scenario is in stark contrast to the circumstances of Gaza patients, where the government financially covers vital treatment protocols. However, despite this coverage, financial challenges persist as barriers in pre-diagnosis and post-treatment procedures, necessitating patients to bear certain costs. This situation also introduces uncertainty regarding when the treatment will become available and accessible because the pathway of patients is not predictable but subjected to approvals in each stage by different authorities, medical and financial approval by the Palestinian MoH, and security approval by Israeli authorities. This is quite a unique context of shortages of medications and barriers to access care compared to other contexts but well documented by health and human rights organisations working in the oPt [1,15].

### 4.2 Surviving in every possible way upon facing life-threatening facts

Cancer treatment delays are a critical global health issue, with measurable impacts on mortality. Studies show that even a four-week delay can increase mortality across various treatment types—surgery, systemic treatments, and radiotherapy—for seven different cancer types [24]. While earlier studies have indicated that factors such as lack of employment, healthcare insurance, and social support can impact women’s adherence to cancer treatment [25], the scenario appears different for BC patients in this study. Participants demonstrate a remarkable commitment to commence treatment as soon as it becomes available or accessible to them. Often, they are faced with less favourable treatment options or the need to bear a financial burden to minimise waiting times. This somehow jeopardizes the application of the concepts of acceptability and quality as per the core components of the RTH framework. However, these challenges do not typically lead to a delay in the initiation of treatment and demonstrate that acceptability and quality play a marginal role in the treatment cycle due to the limited options available to the patients. This underscores that, in the context of Gaza, the determination to survive and pursue treatment promptly, despite various obstacles, remains undiminished. This differed from the compliance pattern among cancer patients in the emergency context who had free access to screening and treatment services yet reported a high non-compliance rate [26].

### 4.3 Cancer care at the margin like never before

In oPt, as an under-resourced country, there is a lack of adequate healthcare system infrastructure to support comprehensive multidisciplinary BC care [27]. This study’s data collection preceded an initiative to establish a Gaza cancer care centre for adults, aiming to provide a strategic vision for optimal cancer care. However, this hope was short-lived. The hospital ceased operations in October 2023 due to a fuel shortage and subsequently faced multiple airstrikes, leading to its complete shutdown [28]. The failure to protect healthcare provision and ensure access has become evident not only through the marginalization of Non-Communicable Diseases and the collapse of healthcare systems but also through the deprivation of essential services such as treatment, food, and water [29]. While human rights protections are enforceable in states with established and advanced governance, they fall short in a nation lacking self-determination. The absence of political will and instability disproportionately harms those in need. In such contexts, validating health-seeking behaviour becomes almost irrelevant. Those who do not succumb to illness might fall victim to bombardment or starvation. People are preoccupied with survival, escaping fear and horror, rather than attending to their health needs. All cancer patients in Gaza have lost access to care. Although specific reporting on the number of deaths remains unavailable at the time of writing this paper [30], many cancer patients were reported dead [31], and the dire situation underscores the critical importance of respecting and upholding international humanitarian and human rights laws, especially in conflict zones, to protect all civilians, with special care to those in need of medical care. The failure to maintain stable and functional institutional arrangements will hinder predicting care-seeking patterns for any illness. Not only did the Health Belief Model fall short in capturing or predicting the behaviour of Gazan women, but other frameworks that surpassed rational choice and considered social dynamics, such as the Social Organization Strategy Framework, also have limitations in explaining or predict behaviour where the institutional arrangements are fragile. The social organization strategy framework [32] introduced by Pescosolido in 1992 is a dynamic, network-centered, and event-based approach that examines decision-making processes. It views decision-makers as social and pragmatic actors engaged in patterned interactions within ongoing relational dynamics – social networks in a broader macro-variable context. While this framework effectively elucidates the nuances and variations in behaviour across experiences of the same patients or among different patients, It does encounter limitations in adequately addressing societal institutional disruptions, dysfunction, or collapse, as the case in this paper. This aligns with literature on the challenges of cancer care in conflict-affected regions, where inequity in access to diagnosis and treatment reflects significantly higher mortality rates among vulnerable populations in Arab conflict zones [33].

## 5 Conclusion

The paper presented the main structural categories that shape health-seeking behaviour among women diagnosed with BC, with these categories serving as either facilitators or hindrances in the pursuit of medical care. Regarding *access* to healthcare, some women promptly sought consultations upon the onset of symptoms, while others prioritised social and work commitments, leading to delayed medical care. Concerning the (un)*availability* of healthcare, women consistently pursued necessary treatment post-confirmation of diagnosis, but if follow-up measures incurred out-of-pocket expenses, they tended to skip them. Regarding the *acceptability* of health services, life-saving procedures were promptly undertaken, irrespective of personal preferences or structural barriers. In the *quality* aspect of the treatment journey, commitment to treatment protocols remained intact with the persistent gaps in availability. However, delayed visits related to side effects, complications, and follow-ups occurred due to low expectations in alleviating endured pain.

Nevertheless, the entire model of health-seeking behaviour or RTH may be deemed less relevant in unstable, conflict-affected contexts where grave violations of rights are committed with impunity. In such high escalation periods, the needs of cancer patients are not prioritised amidst the higher focus on trauma casualties and overall survival priorities. On the other hand, during protracted emergencies —occupation and blockade with less intensity of military attacks—barriers to seeking health care often excel over the seeking action itself.

## 6 Recommendations

Amidst the challenges facing Gaza’s public healthcare system and the recent critical events since October 7, 2023, which have led to severe destruction of the health system and civil society, disrupting previous arrangements, we emphasise the importance of prioritising cancer-related interventions. This is crucial not only in the post-conflict period but also during prolonged conflicts, especially amidst emerging priorities. NGOs must closely collaborate with the government to address the pressing healthcare needs of the population. The RTH as a universal right for all should be enforced even during wartime, ensuring that no human is deprived of their rights to food, water, and medical care. These rights must be protected, respected, and fulfilled by all duty-bearers on a non-discriminatory basis.

The examination of seeking behaviour within the current healthcare structures reveals a dire situation for women with cancer in Gaza. They lack access to essential needs and treatment, raising concerns about prioritising Non-Communicable Diseases care during severe conflict. The unique challenges in Gaza include the absence of alternatives, the failure to implement normative frameworks, and the collapse of local healthcare structures. The forced shutdown of the sole cancer centre and evacuation of the hospital team, along with border closures, necessitate the activation of an urgent referral mechanism for cancer patients with unconditional access to needed services to neighbouring countries until the cancer centre can resume operations.

In a more stable situation with access to essentials like food, water, electricity, and internet services, recommendations can focus on enhancing patient education about their medical conditions and exploring potential remote/online management of cases during escalations. Implementing internal emergency referral procedures could contribute to maintaining the health status of patients. However, before addressing these aspects, all duty-bearers must ensure safe shelters and provide access to all necessary services, including palliative care.

In a scenario before October 7, prioritising the expansion of insurance coverage to alleviate financial burdens, especially for additional expenses incurred by impoverished patients, and considering social security schemes for ill women should also be emphasised. Ensuring the continuous availability of local treatment is a primary priority to avoid delays and uncertainties associated with treatment journeys.

## 7 Limitations

The study presents the outcome of the experiences of Palestinian women living in the GS, which is not necessarily generalisable to Palestinian women living in the WB, who were not feasible to include in the study due to geographical segregation. Institutional factors differ from region to region. There are disparities between Gaza and the WB regarding accessibility and availability of health services, with the WB experiencing better conditions after the political-Palestinian divide started in 2007.

Moreover, with the current aggression on Gaza, all institutional and structural arrangements have been dismantled. The situation of these women requires special care as they find themselves in tents under constant risk, necessitating a follow-up study on the emerging needs and complications arising from the unavailability of basic health needs.

Nevertheless, external researchers and social scientists have limitations in accessing Gaza and interacting with patients due to geographical isolation. The findings can serve as valuable guidance for policymakers, enabling them to develop and implement programs that enhance underlying determinants of survivors’ experiences and well-being.

## Supporting information

S1 Checklist Inclusivity in global research. (DOCX)

## References

[1] AlWaheidi S. Promoting cancer prevention and early diagnosis in the occupied Palestinian territory. Journal of Cancer Policy. 2023;35:100373. 36493987 10.1016/j.jcpo.2022.100373

[2] Ministry of Health - MoH. Health Annual Report, Palestine 2021. MoH: Ramallah, Palestine;2022.

[3] World Health Organization – WHO. Health conditions in the occupied Palestinian territory, including east Jerusalem, and the occupied Syrian Golan. WHO; 2023. https://apps.who.int/gb/ebwha/pdf_files/WHA76/A76_15-en.pdf

[4] World Health Organization – WHO. Health Access Barriers for patients in the occupied Palestinian territory. WHO; 2023. https://www.emro.who.int/images/stories/July_2023_Monthly_2.pdf?ua=1

[5] Bouquet B, Barone-Adesi F, Lafi M, Quanstrom K, Riccardi F, Doctor H, et al. Comparative survival of cancer patients requiring Israeli permits to exit the Gaza Strip for health care: A retrospective cohort study from 2008 to 2017. Plos One. 2021;16(6):e0251058. doi: 10.1371/journal.pone.0251058 34077436 PMC8172025

[6] World Health Organization. Gaza Health Access 2022. WHO; 2023. https://www.emro.who.int/images/stories/palestine/finalGaza_Health_Access_2022_infographic_002.pdf?ua=1

[7] Panato C, Abusamaan K, Bidoli E, Hamdi-Cherif M, Pierannunzio D, Ferretti S, et al. Survival after the diagnosis of breast or colorectal cancer in the GAZA Strip from 2005 to 2014. BMC Cancer. 2018;18(1):632. doi: 10.1186/s12885-018-4552-x 29866055 PMC5987449

[8] World Health Organization – WHO. oPt Emergency Situation Update – issue 21. WHO; 2024. https://www.emro.who.int/images/stories/Sitrep_-_issue_21B.pdf?ua=1

[9] UNFPA. Pathway to Survival - the Story of Breast Cancer in Palestine. UNFPA; 2018. https://palestine.unfpa.org/en/publications/pathway-survival-story-breast-cancer-palestine

[10] Elshami M, Abu Kmeil H, Abu-Jazar M, Mahfouz I, Ashour D, Aljamal A, et al. Breast Cancer Awareness and Barriers to Early Presentation in the Gaza-Strip: A Cross-Sectional Study. J Glob Oncol. 2018;4:1–13. doi: 10.1200/JGO.18.00095 30372400 PMC7010447

[11] Oberoi S, Chaudhary N, Patnaik S, Singh A. Understanding health seeking behavior. J Family Med Prim Care. 2016;5(2):463–4. doi: 10.4103/2249-4863.192376 27843863 PMC5084583

[12] Ammar-Shehada W, Bracke P. The influence of socio-demographic factors on the stage at which women’s breast cancer is diagnosed and treatment prescribed in the Gaza Strip, occupied Palestinian territory. ecancermedicalscience. 2023;17. 10.3332/ecancer.2023.1522 PMC10129395 37113708

[13] Janz NK, Becker MH. The health belief model: a decade later. Health Educ Q. 1984;11(1):1–47. doi: 10.1177/109019818401100101 6392204

[14] UN Economic and Social Council. General Comment No. 14: The Right to the Highest Attainable Standard of Health (Art. 12 of the Covenant. UN Committee on Economic, Social and Cultural Rights (CESCR), 2000 [cited February 8 2024]. https://www.refworld.org/legal/general/cescr/2000/en/36991

[15] World Health Organization – WHO. Right to Health: Barriers to health and attacks on health care in the occupied Palestinian territory, 2019-2023. WHO; 2023. https://applications.emro.who.int/docs/9789292740887-eng.pdf

[16] World Health Organization and United Nations Human Rights Office of the High Commissioner. A human rights-based approach to health. WHO and UNHCHR; 2021. https://www.ohchr.org/sites/default/files/HRBA_HealthInformationSheet.pdf

[17] Kvale S, Brinkmann S. Interviews: Learning the craft of qualitative research interviewing. Sage; 2009.

[18] The United Nations Relief and Works Agency for Palestine Refugees in the Near East - UNRWA. Unrwa in gaza - key statistics. UNRWA; 2023. https://www.unrwa.org/gaza-emergency

[19] Goldsmith LJ. Using framework analysis in applied qualitative research. Qualitative Report. 2021;26(6).

[20] Penchansky R, Thomas JW. The concept of access: definition and relationship to consumer satisfaction. Med Care. 1981;19(2):127–40. doi: 10.1097/00005650-198102000-00001 7206846

[21] Agatha Ogunkorode RevSr, Holtslander L, Ferguson L, Maree JE, Anonson J, Ramsden VR. Factors influencing the health-seeking behaviors of women with advanced stages of breast cancer in Southwestern Nigeria: An interpretive description study. International Journal of Africa Nursing Sciences. 2021;14:100273. doi: 10.1016/j.ijans.2020.100273

[22] National Cancer Institute. Financial Toxicity and Cancer Treatment (PDQ®)–Health Professional Version. n.d. 27583328

[23] Salisu W, Mirlashari J, Varaei S, Seylani K. Limited access to care for persons with breast cancer in Africa: A systematic review. European Journal of Oncology Nursing. 2021;50:101867. 33276292 10.1016/j.ejon.2020.101867

[24] Hanna T, King W, Thibodeau S, Jalink M, Paulin G, Harvey-Jones E, et al. Mortality due to cancer treatment delay: systematic review and meta-analysis. BMJ. 2020;371. 10.1136/bmj.m4087 PMC7610021 33148535

[25] Balkrishnan R, Batten G, Desai R, Daniels Z, Novruzov L, Mole S, et al. Examining predictors of female cancer treatment delay in Appalachian women. n.d.

[26] Sayan M, Eren M, Kötek A, Kaplan S, Duran Ö, Çukurçayır F, et al. Utilization of radiation therapy and predictors of non-compliance among Syrian refugees with cancer living in Turkey. International Journal of Radiation Oncology, Biology, Physics. 2020;108(3):S135.

[27] El Saghir NS, Adebamowo CA, Anderson BO, Carlson RW, Bird PA, Corbex M, et al. Breast cancer management in low resource countries (LRCs): consensus statement from the Breast Health Global Initiative. The Breast. 2011;20(S3):S3-11. 21392996 10.1016/j.breast.2011.02.006

[28] Action on Armed Violence. Gaza healthcare crisis: urgent action required to address alleged unlawful Israeli attacks. AOAV; 2023. https://reliefweb.int/report/occupied-palestinian-territory/gaza-healthcare-crisis-urgent-action-required-address-alleged-unlawful-israeli-attacks

[29] Office of the High Commisioner for Human Rights. Conflict and crisis expose failure to advance the right to health. 2023. https://www.ohchr.org/en/opinion-editorial/2023/12/conflict-and-crisis-expose-failure-advance-right-health

[30] Arab Center Washington DC. “An Open-Air Graveyard”: The Unfolding Health Catastrophe in the Gaza Strip. 2023. https://arabcenterdc.org/resource/an-open-air-graveyard-the-unfolding-health-catastrophe-in-the-gaza-strip/

[31] Euro-Med Human Rights Monitor. Gaza: Palestinian cancer patients die due to hospital closures, lack of medical care. 2023. https://euromedmonitor.org/en/article/5926/Gaza:-Palestinian-cancer-patients-die-due-to-hospital-closures,-lack-of-medical-care

[32] Pescosolido BA. Beyond Rational choice: the social dynamics of how people seek help. American Journal of Sociology. 1992;97(4):1096–138. doi: 10.1086/229863

[33] Al-Shamsi HO, Abu-Gheida IH, Iqbal F, Al-Awadhi A. Cancer in the Arab world. Springer Nature; 2022.

